# Surgical treatment in oligometastatic breast cancer

**DOI:** 10.3332/ecancer.2019.931

**Published:** 2019-05-20

**Authors:** Catalina Vargas, Cristóbal Maiz, María Elena Navarro, David Oddó, César Sánchez, Marisa Bustos, Mauricio Camus

**Affiliations:** 1 Department of Surgical Oncology, Pontificia Universidad Católica de Chile, Santiago 8331150, Chile; 2 Department of Radiology, Pontificia Universidad Católica de Chile, Santiago 8331150, Chile; 3 Department of Anatomical Pathology, Pontificia Universidad Católica de Chile, Santiago 8331150, Chile; 4 Department of Hematology-Oncology, Pontificia Universidad Católica de Chile, Santiago 8331150, Chile; 5 Departament of Oncological Radiotherapy, Pontificia Universidad Católica de Chile, Santiago 8331150, Chile

**Keywords:** metastatic breast cancer, stage IV breast cancer, surgery, locoregional treatment, oligometastatic

## Abstract

Metastatic breast cancer (MBC) management is based on systemic treatment (ST), while the local therapy role remains controversial. We present the case of a 36-year-old woman with a diagnosis of hormone receptor-positive and human epidermal growth factor receptor type 2-positive breast cancer and isolated sternal metastasis, who received neoadjuvant ST with complete remission and later primary tumour surgery. Oligometastatic patients are a subgroup of MBC that can benefit from aggressive local therapies, even with curative intent.

## Introduction

Metastatic breast cancer (MBC) is considered an incurable disease; however, significant improvements in the survival of these patients have been reported with the introduction of new systemic treatment (ST). For this reason, the surgery that was only indicated for palliation has begun to be reconsidered for its therapeutic purposes [1]. The objective of this work is to present the case of a patient with oligometastatic breast cancer (OBC) treated with neoadjuvant ST and primary tumour surgery, and to discuss the role of surgery in patients with MBC, the impact of locoregional treatment (LT) on survival and the concept of oligometastatic disease as a subgroup that can benefit particularly from surgery.

## Case report

**Figure 1.**
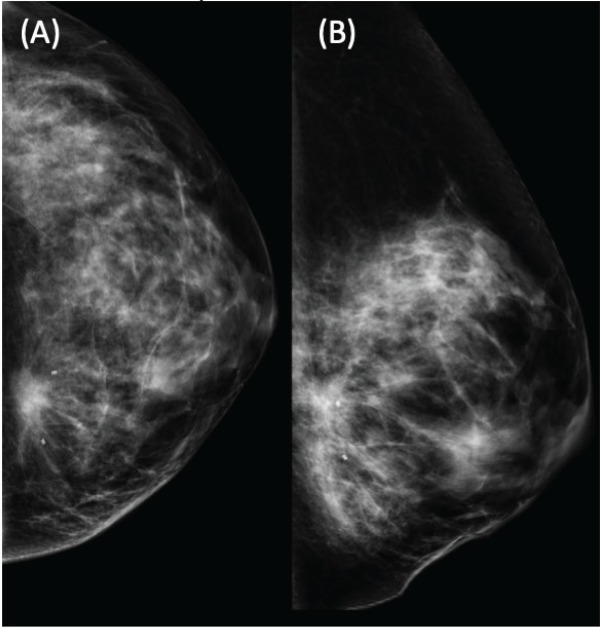
Left mammography showing a dense mammary pattern, with a heterogeneous spiculated dense mass located in the LIQ. BIRADS-5. Two clips marking the lesion prior to neoadjuvant treatment. (A) Craniocaudal and (B) mediolateral oblique view.

**Figure 2.**
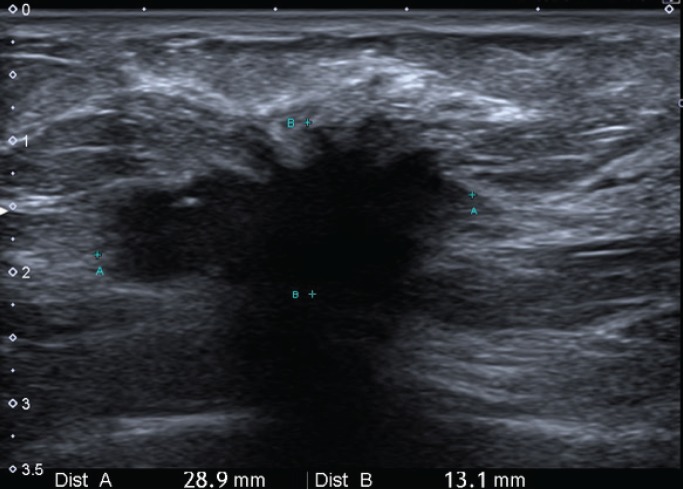
Ultrasonic left breast showing a hypoechogenic-spiculated mass with the acoustic shadow of 29 × 24 × 14 mm3 in correspondence with the mammographic image.

**Figure 3.**
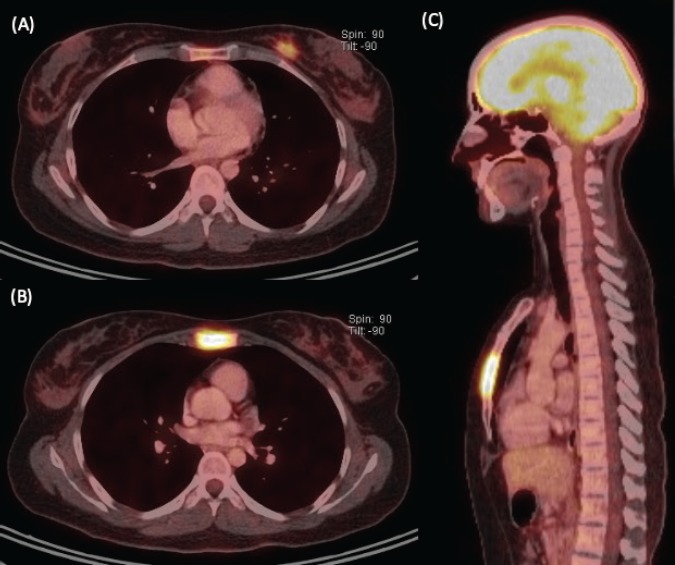
Staging image study. (A) Axial view of PET/CT showing a hypermetabolic left breast nodule of 18 mm at the LIQ, compatible with primary neoplasia. (B) Axial and (C) sagittal views of PET/CT demonstrating extensive substitutive hypermetabolic sternal compromise with cortical osteolysis (maximum SUV 7.7).

**Figure 4.**
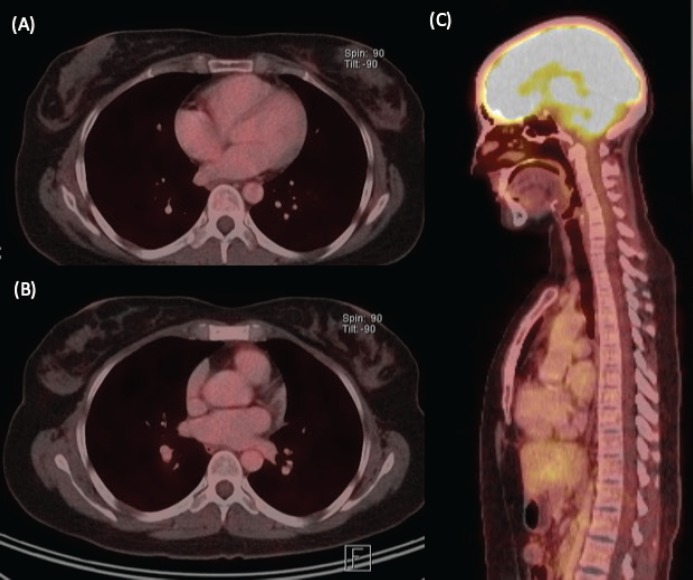
Re-staging imaging study, after 6 months of ST. (A) Axial view of PET/CT with size decrease and hypermetabolism resolution at the left breast nodule. (B) Axial and (C) sagittal views of PET/CT showing hypermetabolism resolution at the sternal body lesion.

We present the case of a 36-year-old woman, with insulin resistance, without a family history of breast cancer, who consulted for a 6-month evolution self-detected left breast nodule associated with skin retraction. At the physical exam, there was a 20-mm palpable mass retracting the skin at the lower-inner quadrant (LIQ) of the left breast, without clinically evident axillary adenopathies. The patient was studied with mammography [Figure 1] and breast ultrasound [Figure 2], which showed a spiculated mass of 29 × 24 × 14 mm at the LIQ of left breast, with the increased flow at doppler, diagnosed as BIRADS-5. A core biopsy was performed, demonstrating a poorly differentiated invasive ductal carcinoma with positive oestrogen receptor (99%; ++/+++), positive progesterone receptor (90%; +++), positive human epidermal growth factor receptor type 2 (HER2) 3+, fluorescence *in situ* hybridisation for HER2+ and Ki-67 30%. The staging study demonstrated an osteolytic lesion compatible with sternal metastasis evident in computed tomography (CT), positron emission tomography (PET)/CT and magnetic resonance imaging (MRI) [Figure 3]. The staging was completed with sentinel lymph node biopsy, obtaining six lymph nodes without metastasis. The primary lesion was marked with two metallic clips guided by ultrasound. The patient completed neoadjuvant ST with Doxorubicin, Cyclophosphamide, Paclitaxel, and HER2-directed therapy with Trastuzumab and Pertuzumab, with a complete clinical response at physical exam and images [Figure 4]. Later, she underwent left partial mastectomy, using percutaneous hookwire for the location of the metallic marker clips. The definitive biopsy demonstrated an area of 27 × 25 × 15 mm of scarring substitutive fibrosis associated with isolated microfocus (less than 1 mm) of moderately differentiated invasive ductal carcinoma with negative margins. The study of residual tumour load reported 1% invasive carcinoma and 0% intraductal carcinoma *in situ*. Two months after the surgery, LT with radiotherapy was completed. The breast was treated with tangential X-ray fields of 6 and 18 MV, the supraclavicular and left axillary region with a right anterior oblique field, and the internal mammary territory and sternum with an array of photons of 18 MV and electrons of 9 MeV, completing in all areas a dose of 50 Gy in 25 fractions in 5 weeks and then a boost of 10 Gy in 5 fractions over the tumour bed. Subsequently, it was decided to complete ST with Trastuzumab and Pertuzumab, in addition to pharmacological ovarian suppression with Triptorelin plus Tamoxifen. The HER2-directed therapy will be maintained until the progression of the disease or toxicity.

## Discussion

MBC is defined by the presence of distant metastases, most commonly to the bone, lung, liver and brain. It corresponds approximately to 6% of newly diagnosed breast cancer in the USA each year, being associated with worse prognosis, with a median survival of 2 years for women diagnosed as stage IV *de novo* [1]. MBC management is based on ST, reserving LT (surgery or radiotherapy), for symptoms palliation and management of complications such as skin ulceration, bleeding, pain and the presence of a fungating mass [2].

### Impact of locoregional treatment on survival

The impact of surgical resection of the primary tumour on survival in metastatic patients is controversial. A series of retrospective studies on LT of the novo diagnosed stage IV breast cancer suggests a survival benefit in those patients who underwent complete resection of the primary tumour; however, those studies present important selection bias of the subjects. Included patients submitted to surgery used to be younger, with solitary or few metastases, less tumour burden and better response to ST, which probably influenced the results [3–4].

One multicenter prospective study assessed the role of surgery on stage IV breast cancer. The translational breast cancer research consortium 013 [5], enrolled 112 patients with *de novo* MBC diagnosed between 2009 and 2012, all who received first-line ST, and the patients who responded underwent surgery. Eighty-five per cent of patients were classified as responders, with significantly higher survival at 3 years when compared to non-responders (78% versus 24%, *p* < 0.001). Between responders, 41% were submitted to surgery, without impact on global survival at 3 years (77% with surgery versus 76% without surgery). These patients had bigger tumours, metastasis isolated in one organ and first-line chemotherapy.

Two randomised clinical trials (RCTs) have assessed LT for *de novo* MBC. An RCT in the Tata Memorial Centre, India [6], randomised 350 patients between 2005 and 2013 (14 with resectable hormone receptor-positive primary tumours and 336 with unresectable primary tumours treated with neoadjuvant first-line chemotherapy, with partial or complete response) to LT (*n* = 173) and no-LT (*n* = 177), without a significant improvement in global survival at 2 years (41.9% versus 43%, respectively). It is worth mentioning that 10% of patients without LT required palliative surgery. Systemic chemotherapy did not include taxanes uniformly; 92% of HER2 (+) patients did not receive HER2-directed therapy and patients with isolated metastasis who were good candidates for curative treatment were excluded from the study. In an RCT conducted in Turkey, the MF07-01 [7] randomised 274 patients between 2007 and 2012, to LT plus ST (*n* = 138) or only ST (*n* = 136), without differences in global survival at 3 years (60% versus 51%, respectively, *p* = 0.10), finding a 34% decrease in risk of death in the LT group at 40 months (HR 0.66; 95% CI: 0.49–0.88, *p* = 0.005). Between the no-LT group, 5.9% required palliative surgery and stratification of factors before randomisation was not planned, resulting in a higher rate of hormone receptor-positive tumours and a lower rate of triple-negative tumours in the LT group (85.5% versus 71.8% and 7.3% versus 17.4%, respectively), which could have influenced the results. Moreover, at the subgroup analysis it was observed that triple-negative tumours had no benefit from surgery, showing 85% mortality at 17.5 months, compared with 18 months without surgery, and the patients with multiple hepatic or pulmonary metastases had a significantly worse prognosis with surgery at 3 years (survival of 31% versus 67% without surgery). Both RCTs have limitations and their results cannot be extrapolated to centres where patients with the advanced disease receive neoadjuvant ST with anthracycline/taxane-based chemotherapy and HER2-directed therapy (Trastuzumab and/or Pertuzumab) when indicated. Two RCTs are underway to clarify the current controversies: the Eastern Cooperative Group 2108 and the Japan Clinical Oncology Group 1017 [1]. Until the results of these studies are available, it is not recommended to patients with *de novo* stage IV breast cancer to undergo surgery with the aim of improving survival.

### Oligometastatic disease

MBC is a heterogeneous disease, which includes both single metastatic lesions and diffuse involvement of multiple organs. The term oligometastases, coined in 1995 by Hellman and Weichselbaum [8], refers to a metastatic disease characterised by only one or few detectable metastases, usually less than 5 and smaller than 5 cm. It is proposed that the oligometastatic disease corresponds to an intermediate clinical state, with a limited number and locations of metastases, representing an early stage in the chain of metastatic progression amendable through the application of a curative therapeutic strategy. The incidence of OMB is unknown, and it is estimated at 1%–10% of newly diagnosed metastatic disease, but it could increase as imaging studies such as PET/CT and MRI become more accessible, improving the ability to detect metastasis. Several retrospective studies show that OBC patients have a better prognosis when are treated with aggressive LT, reaching overall survival rates of 82% at 10 years and 53% at 20 years, but it is not clear if it is due to more favourable tumour biology or to the effects of treatment [9].

## Conclusion

MBC cancer is a systemic disease and there is a lack of evidence to show that LT improves the survival of these patients. Because of the above, and given that breast cancer stage IV includes a heterogeneous spectrum of pathologies, primary tumour surgery should be considered judiciously, being able to have both palliative and therapeutic purpose, depending on the burden of disease and the response to ST of each patient, with situations like oligometastatic disease becoming potentially curable.

## Informed consent

The patient gave her informed consent to publish the case.

## Conflicts of interest

The authors have no financial interest or any conflicts of interest.
